# Novel pathogenic variants and multiple molecular diagnoses in neurodevelopmental disorders

**DOI:** 10.1186/s11689-019-9270-4

**Published:** 2019-06-25

**Authors:** Joanne Trinh, Krishna Kumar Kandaswamy, Martin Werber, Maximilian E. R. Weiss, Gabriela Oprea, Shivendra Kishore, Katja Lohmann, Arndt Rolfs

**Affiliations:** 1 Institute of Neurogenetics, University of Lübeck, 23538 Lübeck, Germany; 2 Centogene AG, Rostock, Germany; 3 University of Rostock, 18147 Rostock, Germany

**Keywords:** Neurodevelopmental disorders, De novo variants, Trio exome sequencing

## Abstract

**Background:**

Rare denovo variants represent a significant cause of neurodevelopmental delay and intellectual disability (ID).

**Methods:**

Exome sequencing was performed on 4351 patients with global developmental delay, seizures, microcephaly, macrocephaly, motor delay, delayed speech and language development, or ID according to Human Phenotype Ontology (HPO) terms. All patients had previously undergone whole exome sequencing as part of diagnostic genetic testing with a focus on variants in genes implicated in neurodevelopmental disorders up to January 2017. This resulted in a genetic diagnosis in 1336 of the patients. In this study, we specifically searched for variants in 14 recently implicated novel neurodevelopmental disorder (NDD) genes.

**Results:**

We identified 65 rare, protein-changing variants in 11 of these 14 novel candidate genes. Fourteen variants in *CDK13*, *CHD4*, *KCNQ3*, *KMT5B*, *TCF20*, and *ZBTB18* were scored pathogenic or likely pathogenic. Of note, two of these patients had a previously identified cause of their disease, and thus, multiple molecular diagnoses were made including pathogenic/likely pathogenic variants in *FOXG1* and *CDK13* or in *TMEM237* and *KMT5B*.

**Conclusions:**

Looking for pathogenic variants in newly identified NDD genes enabled us to provide a molecular diagnosis to 14 patients and their close relatives and caregivers. This underlines the relevance of re-evaluation of existing exome data on a regular basis to improve the diagnostic yield and serve the needs of our patients.

**Electronic supplementary material:**

The online version of this article (10.1186/s11689-019-9270-4) contains supplementary material, which is available to authorized users.

## Background

Major congenital malformations, which include neurodevelopmental disorders (NDDs), are present in ~ 2–5% of children [1]. Children with NDD have variable severity of phenotypic features and different behavioral abnormalities. Often times, NDD arises from de-novo variants in genes important for central nervous system (CNS) development [2]. Whole exome sequencing has been critical and effective in diagnosing patients with NDD. Thus, treatment for NDD has become more refined through molecular genetic diagnosis rather than phenotype-driven management of symptoms [3]. Herein, we find novel pathogenic or likely pathogenic variants in six recently identified NDD genes, namely *CDK13*, *CHD4*, *KCNQ3*, *KMT5B*, *TCF20*, and *ZBTB18*.

## Methods

### Patients

From a total of 26,119 in-house exome data set, we included 4351 unrelated NDD patients in this study. Human Phenotype Ontology (HPO) nomenclature [4] was applied based on the clinical data provided by referring physician. In the context of this manuscript, NDD was defined by HPO terms described in Additional file 1: Figure S1. Patients had an average age of 7.75 (STD 8.04) years (Additional file 1: Table S1). All patients had previously undergone whole exome sequencing as part of their clinical genetic testing, following previously reported procedures [5]. These tests focused on NDD genes established before January 2017. Parents were available from 2030 patients to test for de novo occurrence of variants. Written informed consent was obtained from participants, and this study was approved by the Ethical Commission of the University of Rostock (registry no. A2015-0102). All samples were processed in Centogene’s laboratory, which is CAP and CLIA certified, adhering to the American College of Medical Genetics and Genomics (ACMG) guidelines [6].

### Genetic testing

Patient DNA was extracted from EDTA blood or from dry blood spots in filter cards. WES was performed on the IonProton (*n* = 911 samples, enrichment with Ion AmpliSeq Exome RDY Kit (Life Technologies, Carlsbad, CA, USA)) or Illumina (*n* = 3440 samples, enrichment with Illumina’s NexteraRapid Capture Exome Kit (Illumina, Inc., San Diego, CA, USA)). Sequencing and bioinformatics were done as previously described [5, 7, 8]. We focused on genes of interest (fourteen recently nominated genes by the DDD study [9]; Additional file 1: Figure S1), filtered for rare variants (MAF < 0.0001), and an effect on the encoded protein sequence. Sanger validations were performed for all indels and variants with quality Phred score below 300 to rule out false-positive variants as previously described [5]. Further, we applied the ACMG criteria to score the pathogenicity of candidate variants [6].

## Results

**Fig. 1:**
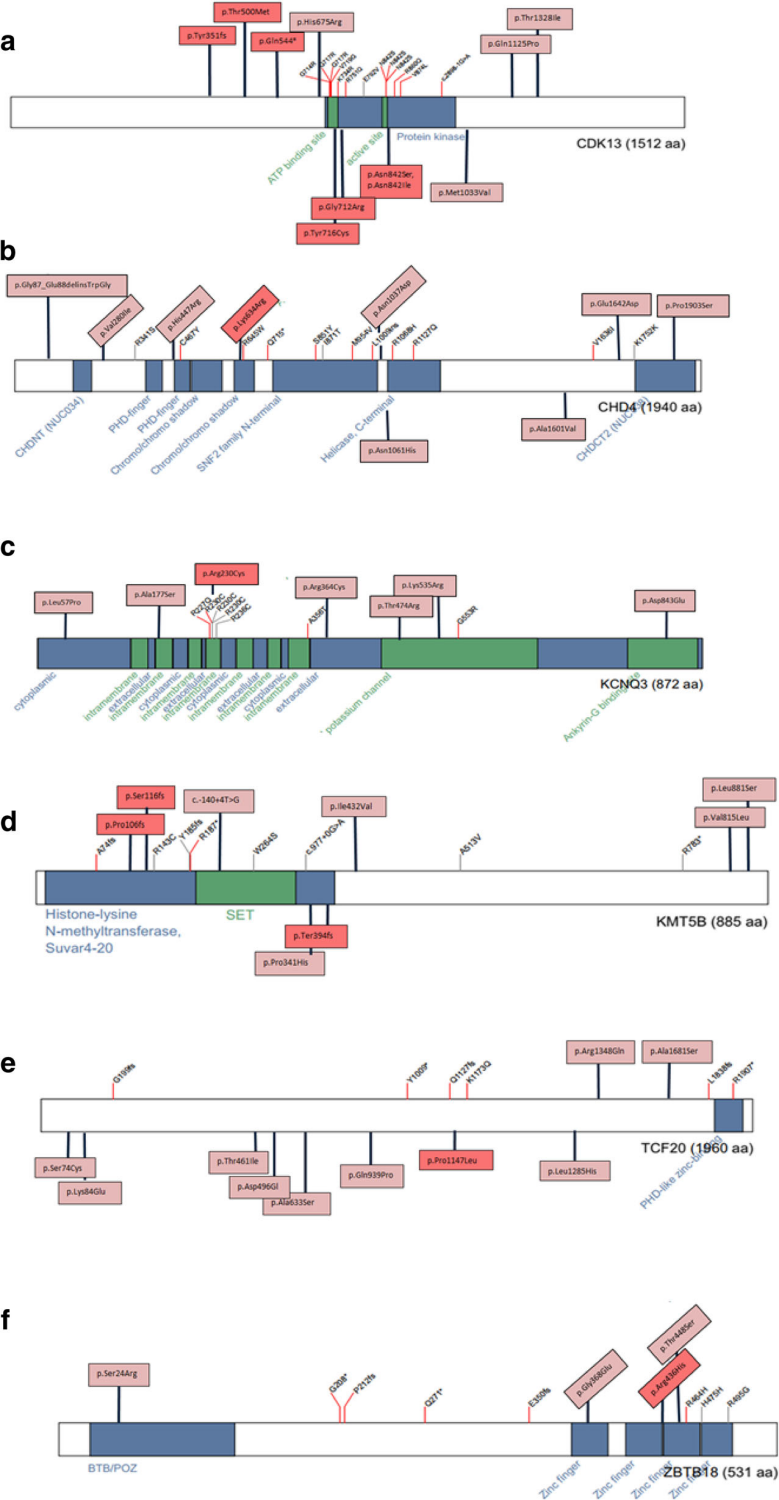
**a**-**f** Composite figures of genes with pathogenic or likely pathogenic variants identified in this study: *CDK13*, *CHD4*, *KCNQ3*, *KMT5B*, *TCF20*, and *ZBTB18* (adapted from the “Prevalence and architecture of de novo mutations in developmental disorders” study [9]). Boxes: pink highlighted variants are VUS and red highlighted variants are pathogenic or likely pathogenic changes. Functional domains of the encoded protein are indicated by blue boxes. Variants that have already been identifeid in the previous study are shown with red branching

Among all 4351 NDD patients, we identified 65 heterozygous variant carriers (1.5%), for 65 different rare, protein sequence-changing variants in 11 out of 14 genes recently nominated by the DDD study [9] (Additional file 1: Figure S1 and Table S2). In 11 of 12 carriers for whom parents were available, the variant was shown to be de novo, and in one case (*KCNQ3*:p.Arg364Cys) inherited from the father whereby his affection status is unknown. The variant *CDK13*: p.His675Arg was found in two affected siblings. For all other patients, no relatives were available for testing. The 65 variants were either not present or at very low frequency (< 2.76 × 10^− 4^ frequency) in unaffected “in-house” exomes or in public databases (ExAC, GnomAD). Using ACMG recommendations, six of these 65 variants were scored as pathogenic (*CDK13:*p.Tyr351fs, *CDK13:*p.Gln544*, *CDK13:*p.Asn842Ser, *KMT5B:*p.Pro106fs, *KMT5B:*p.Ser116fs, and *KCNQ3:*p.Arg230Cys) and eight as likely pathogenic (*CDK13:*p.Thr500Met, *CDK13:*p.Asn843Ile, *CDK13:*p.Gly712Arg, *CDK13:*p.Tyr716Cys, *CHD4:*p.Lys634Arg, *KMT5B:*p.Ter394fs, *ZBTB18:*p.Arg436His, and *TCF20:*p.Pro1147Leu) (Table 1). The remaining 51 variants (78%) were categorized as variants of uncertain significance (VUS) (Additional file 1: Table S2 and S5; Fig. 1). This included a de novo splice region variant in *KMT5B* (c.-140+4T>G) which was predicted in silico (using HumanSplicingFinder and MaxEntScan) to results in alternative splicing for transcript NM_001300907.1. However, a fresh sample from this patient was not available to test for alterations in splicing. Patients’ clinical characteristics were compared across *CDK13* and *KMT5B* variant carriers (Additional file 1: Figure S2 and S3).

**Table 1:**
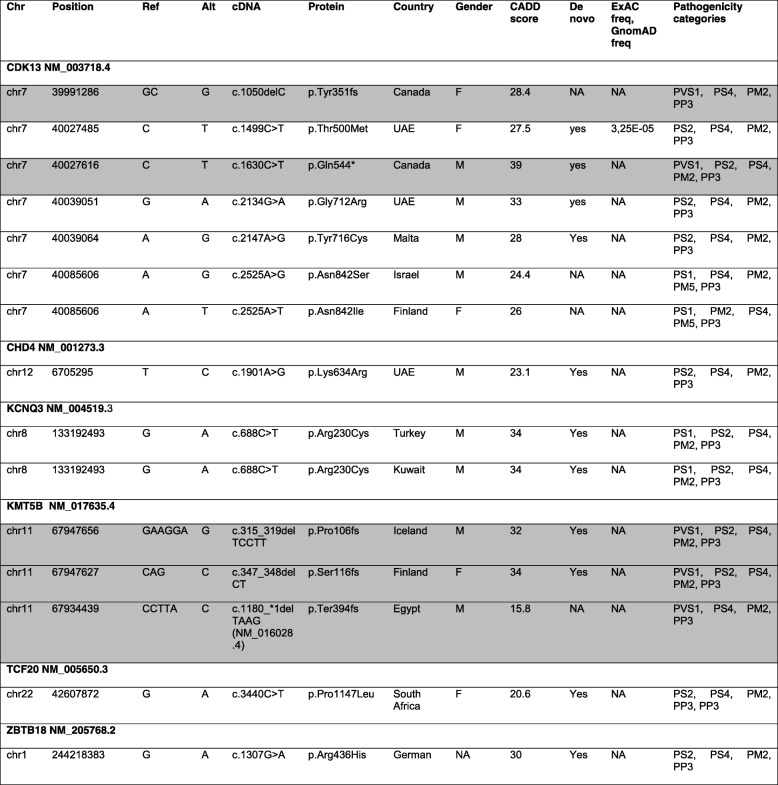
List of pathogenic or likely pathogenic variants in this study

*Chr* chromosome, *Ref* reference allele, *Alt* alternate allele, *De novo* parents available to confirm de novo status, *UAE* United Arab Emirates, *SA* Saudi Arabia. Table includes individual variants from 4351 unrelated patients. Pathogenic variants are shaded in gray and likely pathogenic variants are unshaded

There were two patients who had previously received a genetic diagnosis and thus carried an additional pathogenic variant in a previously established NDD gene (Additional file 1: Table S3). Thus, these two patients each carried multiple molecular diagnoses. This included a patient with a frameshift variant in *FOXG1* (OMIM number 613454) and a missense change in *CDK13* (OMIM number 603309) who had a complex phenotype beyond typical Rett-like syndrome presentation including MRI abnormalities and visual impairment. This patient also had delayed motor and language development, intellectual disability, muscular hypotonia, microcephaly, ventricular septal defect, failure to thrive and squint which aligns with the OMIM phenotype of congenital heart defects, dysmorphic facial features, and intellectual developmental disorder (CHDFIDD). The onset was at birth, and her parents were non-consanguineous, and there were no other affected siblings.

Another patient carried a homozygous c.869+1G>A variant in *TMEM237* (OMIM number 614424) and a frameshift variant c.1180_*1delTAAG (p.Ter394fs) in *KMT5B* (OMIM number 617788). This male patient has been suspected to be affected with Joubert syndrome which is known to be linked to biallelic *TMEM237* variants, and had defective vision and global developmental delay. Whether there is an additional contribution of the likely pathogenic *KMT5B* variant to the phenotype is difficult to determine, although some features overlap with the OMIM phenotype of mental retardation.

## Discussion

In this study, we identified pathogenic/likely pathogenic variants in 14 NDD patients in six different, recently identified genes. Our findings highlight the importance of reanalyzing and revisiting exome sequencing data to reclassify variants of uncertain significance by taking into account novel observations published in the scientific literature. Since the initial study [9], 13 of the 14 investigated genes, with the exception of MSL3, have independently been replicated [10–23] including *CDK13*, *CHD4*, *KCNQ3*, *KMT5B*, *TCF20*, and *ZBTB18*.

In our sample, *CDK13* (cyclin-dependent kinase 13) and *KMT5B* (lysine-specific methyltransferase 5B) harbored the most pathogenic/likely pathogenic variants while the most VUS were detected in *TCF20*. Of note, we found two unrelated patients with a change of the amino acid residue asparagine at position 842 in *CDK13* (p.Asn842Ser and p.Asn842Ile). These patients had delayed speech and language development, motor delay, and abnormal facial shape (Additional file 1: Figure S3 and Table S4). The p.Asn842Ser has also been previously described in the DDD study [9], suggesting that position 842 could be a mutational hot spot.

Notably, there were two patients who carried two pathogenic/likely pathogenic variants in two different genes (*n* = 2/65, 3%) each. Of note, this is in the same range as a recent large-scale study (4.9%) [24], further underlining the importance to search for genetic causes with an exome-wide approach not to overlook relevant genetic diagnoses and also the importance of revisiting and reanalyzing exomes over time as more and more new genetic publications surface, even if one genetic cause has already been identified.

The genetic heterogeneity of NDD with hundreds of genes in which variants lead to NDD reflects the complex process of proper brain development. Many of the gene products function in multiple biological pathways but may result in strikingly different phenotypes. For example, patients with de novo variants in *CDK13* and *CHD4* may present with overlapping neurodevelopmental features and heart defects; the function of both genes is different [9, 25, 26]. CHD4 is part of the SNF2/RAD54 helicase family and is a core component of the nucleosome remodeling and histone deacetylase repressor complex  which is important for epigenetic regulation of gene transcription. In contrast, CDK13 forms a complex with cyclin K and is predicted to have a role in regulating cell cycle but also transcription. On the other hand, a distinct phenotype can be seen for variants within the same gene. *CHD4* somatic variants are also involved in uterine serous carcinoma, an aggressive endometrial cancer [27]. This illustrates the high time and spatial sensitivity of the developing brain/body to genetic variations.

Many novel NDD genes are involved in epigenetic mechanisms such as chromatin remodeling, histone modification, RNA splicing, transcription, and DNA binding including the two most relevant genes from our study, i.e., *CDK13* and *KMT5B.* CDK13 forms a complex with cyclin K and is predicted to have a role in regulating cell cycle and transcription. Mutations can alter complex activity. KMT5B functions as a histone methyltransferases and trimethylate nucleosomal histone 5 [28]. KMT5B also trimethylates the oncogene ERK (extracellular signal-regulated kinases), and overexpression of KMT5B activates the ERK signaling pathway [29]. These kinases are important for brain development, proliferation of cells, and neuronal migration, and ERK1/2 deficits in mice have shown impaired neurogenesis [30]. Histone deacetylase inhibitors (HDACis) and DNA demethylating drugs (DNMTis) have been used in cancer therapy trials [31, 32] and may be emerging drugs in NDD [33].

## Conclusions

Our study underlines the relevance of six additional NDD genes and highlights the significance of multiple genetic diagnoses in several patients. Our study accentuates the importance of re-evaluating whole exome sequencing data in light of new publications enabling reclassification of previously categorized variants of uncertain significance.

## Additional file

Additional file 1: **Table S1.** Patient demographics for developmental disorders. **Table S2**. Variants of unknown significance identified in this study and pathogenicity scoring. **Table S3.** Individuals with dual molecular diagnoses. **Table S4.** HPO terms listed for all (likely) pathogenic mutation carriers. **Table S5.** Number of rare, protein-changing variants found in the NDD patients. **Figure S1.** Overview of study: workflow of identification of 14 (likely) pathogenic variants (6 of 14 candidate genes) in 14 of 4351 patients. **Figure S2.** HPO terms composite for CDK13 pathogenic/likely pathogenic carriers. HPO terms that overlap in different mutation carriers are highlighted in red. **Figure S3.** HPO terms composite for KMT5B pathogenic/likely pathogenic variant carriers. HPO terms that overlap in different mutation carriers are highlighted in red. (DOCX 96 kb)

## Data Availability

All data on variants will be available on HGMD.

## Abbreviations

CDK13: Cyclin-dependent kinase 13
CHD4: Chromodomain-helicase-DNA-binding protein 4
DDD: Deciphering Developmental Disorders
DNA: Deoxyribonucleic acid
EDTA: Ethylenediaminetetraacetic acid
FOXG1: Forkhead box G1
KCNQ3: Potassium voltage-gated channel subfamily Q member 3
KMT5B: Lysine methyltransferase 5B
NDD: Neurodevelopmental disorder
TCF20: Transcription factor 20
TMEM237: Transmembrane protein 237
ZBTB18: Zinc finger and BTB domain containing 18
