# Supplementary material for: Novel pathogenic variants and multiple molecular diagnoses in neurodevelopmental disorders
Source: J Neurodev Disord. 2019 Jun 25;11:11. doi: 10.1186/s11689-019-9270-4 (PMC6593513; doi:10.1186/s11689-019-9270-4)
Supplement: Supplementary file 1 — Table S1. Patient demographics for developmental disorders. Table S2. Variants of unknown significance identified in this study and pathogenicity scoring. Table S3. Individuals with dual molecular diagnoses. Table S4. HPO terms listed for all (likely) pathogenic mutation carriers. Table S5. Number of rare, protein-changing variants found in the NDD patients. Figure S1. Overview of study: workflow of identification of 14 (likely) pathogenic variants (6 of 14 candidate genes) in 14 of 4351 patients. Figure S2. HPO terms composite for CDK13 pathogenic/likely pathogenic carriers. HPO terms that overlap in different mutation carriers are highlighted in red. Figure S3. HPO terms composite for KMT5B pathogenic/likely pathogenic variant carriers. HPO terms that overlap in different mutation carriers are highlighted in red. (DOCX 96 kb) [file 11689_2019_9270_MOESM1_ESM.docx]

**Supplementary Appendix**

| **Table of contents** | **Content** | **Page number** |
| --- | --- | --- |
| Supplementary Table 1 | Patient demographics | 2 |
| Supplementary Table 2 | Variants of unknown significance identified in study and pathogenicity scoring | 3-6 |
| Supplementary Table 3 | Previously identified pathogenic mutations in patients | 7 |
| Supplementary Table 4 | HPO terms listed for all (likely) pathogenic mutation carriers | 8-10 |
| Supplementary Table 5 | List of mutations found in whole dataset of patients with NDD | 11 |
| Supplementary Figure 1 | Overview of study: Workflow of identification of 14 (likely) pathogenic variants in 6 of 14 candidate genes. | 12 |
| Supplementary Figure 2 | HPO terms composite for *CDK13* mutations carriers | 13 |
| Supplementary Figure 3 | HPO terms composite for *KMT5B* mutations carriers | 14 |

Additional file 1: **Table S1.** Patient demographics for developmental disorders

|  | **No. of patients with DD (%)** |
| --- | --- |
| **Geographic region** | |
| *Middle East* | 2904 (66.74) |
| *Europe* | 693 (15.93) |
| *South/North America* | 298 (6.85) |
| *South Asia* | 291 (6.69) |
| *Africa* | 109 (2.51) |
| *Oceania* | 43 (0.99) |
| *Unknown* | 13 (0.3) |
| **Total number of patients** | **4351** |
| Gender ratio | 1.26:1 |
| **Age of patients** | |
| *<1 year* | 594 (13.65) |
| *1-5 years* | 1717 (39.46) |
| *5-15 years* | 1377 (31.65) |
| *15-30 years* | 272 (6.25) |
| *>30 years* | 105 (2.41) |
| *Unknown* | 286 (6.57) |
| **Average age (STD)** | **7.75 (8.04)** |
| **Consanguineous parents** | **1861 (41.07)** |
| **Positive family history** | **1206 (27.71)** |

**DD -** developmental delay, Intellectual disability, Delayed speech and language development, Seizures, Motor delay, Microcephaly or Macrocephaly

Additional file 1: **Table S2**. Variants of unknown significance identified in this study and pathogenicity scoring

| **Chr** | **Position** | **Ref** | **Alt** | **cDNA** | **Protein** | **Country** | **Gender** | **CADD score** | **De novo** | **ExAC freq, GnomAD freq** | **Pathogenicity scoring** |
| --- | --- | --- | --- | --- | --- | --- | --- | --- | --- | --- | --- |
| **CDK13 NM_003718.4** | | | | |  |  |  |  |  |  |  |
| chr7 | 40037245 | A | G | c.2024A>G | p.His675Arg | India | M | 23.2 | NA | NA | PM2, PM6, PP3 |
| chr7 | 40127792 | A | G | c.3097A>G | p.Met1033Val | Lebanon | M | 19.1 | NA | NA | PM2, PM6, PP3 |
| chr7 | 40132522 | AG | CC | c.3374_3375delAGinsCC | p.Gln1125Pro | Iran | M | 23.6 | NA | NA | PM2, PM6, PP3 |
| **CHD4 NM_001273.3** | | | | |  |  |  |  |  |  |  |
| chr12 | 6711300 | CTCCCCA | TCCCCAC | c.258_264delinsGTGGGGA | p.Gly87_Glu88delinsTrpGly | SA | M | 17.6 | yes | NA | PM2, PM6, PP3 |
| chr12 | 6710181 | C | T | c.838G>A | p.Val280Ile | SA | F | 22.9 | NA | NA | PM2, PM6, PP3 |
| chr12 | 6709081 | T | C | c.1340A>G | p.His447Arg | SA | M | 18.9 | NA | NA | PM2, PM6, PP3 |
| chr12 | 6700973 | T | C | c.3109A>G | p.Asn1037Asp | Turkey | M | 25 | NA | NA | PM2, PM6, PP3 |
| chr12 | 6700901 | T | G | c.3181A>C | p.Asn1061His | Kuwait | M | 23.7 | NA | NA | PM2, PM6, PP3 |
| chr12 | 6690317 | G | A | c.4802C>T | p.Ala1601Val | Turkey | F | 23 | NA | NA | PM2, PM6, PP3 |
| chr12 | 6688067 | C | G | c.4926G>C | p.Glu1642Asp | SA | F | 17.1 | NA | NA | PM2, PM6, PP3 |
| chr12 | 6680049 | G | A | c.5707C>T | p.Pro1903Ser | Iran | F | 22.1 | NA | NA | PM2, PM6, PP3 |
| **CNOT3 NM_014516.3** | | |  |  |  |  |  |  |  |  |  |
| chr19 | 54649367 | A | G | c.517A>G | p.Ile173Val | Canada | F | 12.8 | NA | NA | PM2, PM6, PP3 |
| chr19 | 54651971 | C | A | c.983C>A | p.Pro328Gln | India | F | 15 | NA | NA | PM2, PM6, PP3 |
| chr19 | 54651973 | C | G | c.985C>G | p.Pro329Ala | SA | M | 0.9 | NA | NA | PM2, PM6, PP3 |
| chr19 | 54652238 | G | C | c.1250G>C | p.Gly417Ala | Qatar | M | 1.6 | yes | NA | PM2, PM6, PP3 |
| chr19 | 54653357 | A | G | c.1469A>G | p.Asn490Ser | Turkey | M | 0.8 | NA | NA | PM2, PM6, PP3 |
| chr19 | 54656033 | C | T | c.1676C>T | p.Pro559Leu | Iran | F | 32 | NA | NA | PM2, PM6, PP3 |
| chr19 | 54656668 | T | C | c.1969T>C | p.Ser657Pro | India | NA | 21.1 | NA | NA | PM2, PM6, PP3 |
| **KCNQ3 NM_004519.3** | | | |  |  |  |  |  |  |  | PM2, PM6, PP3 |
| chr8 | 133141599 | G | T | c.2529C>A | p.Asp843Glu | Sweden | NA | 20.8 | yes | NA | PM2, PM6, PP3 |
| chr8 | 133150228 | T | C | c.1604A>G | p.Lys535Arg | SA | M | 27.4 | NA | 4,07E-06 | PM6, PP3 |
| chr8 | 133153420 | G | C | c.1421C>G | p.Thr474Arg | India | F | 24 | NA | NA | PM2, PM6, PP3 |
| chr8 | 133184895 | G | A | c.1090C>T | p.Arg364Cys | Canada | M | 34 | NA | 3,23E-05 | PM6, PP3 |
| chr8 | 133196563 | C | A | c.529G>T | p.Ala177Ser | Oman | M | 24.2 | yes | NA | PM2, PM6, PP3 |
| chr8 | 133492610 | A | G | c.170T>C | p.Leu57Pro | Iraq | F | 18.7 | NA | 9,56E-06 | PM6, PP3 |
| **KMT5B NM_017635.4** | | | |  |  |  |  |  |  |  |  |
| chr11 | 67946933 | A | C | c.-140+4T>G (NM_001300907.1) | Unknown | SA | F | 18.6 | yes | NA | PM2, PM6, PP3 |
| chr11 | 67934601 | G | T | c.1022C>A | p.Pro341His | Romania | F | 26.3 | NA | NA | PM2, PM6, PP3 |
| chr11 | 67926519 | T | C | c.1294A>G | p.Ile432Val | Colombia | M | 0.001 | NA | 4,08E-06 | PM6, PP3 |
| chr11 | 67925370 | C | A | c.2443G>T | p.Val815Leu | SA | F | 17.9 | NA | NA | PM2, PM6, PP3 |
| chr11 | 67925171 | A | G | c.2642T>C | p.Leu881Ser | Egypt | F | 26.1 | NA | NA | PM2, PM6, PP3 |
| **MSL3 NM_078629.3** | | | |  |  |  |  |  |  |  |  |
| chrX | 11776876 | C | G | c.50C>G | p.Pro17Arg | SA | F | 7.6 | NA | NA | PM2, PM6, PP3 |
| chrX | 11781106 | G | A | c.739G>A | p.Ala247Thr | Turkey | F | 24.6 | NA | NA | PM2, PM6, PP3 |
| **PUF60 NM_078480.2** | | | |  |  |  |  |  |  |  |  |
| chr8 | 144899286 | C | T | c.1174G>A | p.Val392Ile | Israel | M | 18.4 | yes | NA | PM2, PM6, PP3 |
| chr8 | 144900431 | C | T | c.535G>A | p.Val179Ile | SA | M | 10.7 | NA | NA | PM2, PM6, PP3 |
| **QRICH1 NM_001320580.1** | | | |  |  |  |  |  |  |  |  |
| chr3 | 49067961 | T | C | c.2255A>G | p.Gln752Arg | Brazil | F | 13.9 | NA | NA | PM2, PM6, PP3 |
| chr3 | 49067988 | T | C | c.2228A>G | p.Gln743Arg | SA | F | 25.5 | NA | NA | PM2, PM6, PP3 |
| chr3 | 49094618 | C | T | c.1015G>A | p.Ala339Thr | Turkey | M | 16 | NA | 8,12E-06 | PM6, PP3 |
| chr3 | 49094702 | T | C | c.931A>G | p.Ile311Val | SA | M | 15 | NA | NA | PM2, PM6, PP3 |
| chr3 | 49095086 | C | A | c.547G>T | p.Ala183Ser | Spain | M | 8.8 | NA | NA | PM2, PM6, PP3 |
| **SET NM_001122821.1** | | | |  |  |  |  |  |  |  |  |
| chr9 | 131446275 | C | T | c.101C>T | p.Pro34Leu | Turkey | F | 13.9 | NA | NA | PM2, PM6, PP3 |
| **TCF20 NM_005650.3** | | | |  |  |  |  |  |  |  |  |
| chr22 | 42606271 | C | A | c.5041G>T | p.Ala1681Ser | Turkey | M | 12.6 | NA | NA | PM2, PM6, PP3 |
| chr22 | 42607269 | C | T | c.4043G>A | p.Arg1348Gln | Egypt | M | 33 | NA | 4,06E-06 | PM2, PM6, PP3 |
| chr22 | 42607458 | A | T | c.3854T>A | p.Leu1285His | Iran | F | 22.1 | NA | NA | PM6, PP3 |
| chr22 | 42608496 | T | G | c.2816A>C | p.Gln939Pro | German | M | 20.6 | NA | NA | PM2, PM6, PP3 |
| chr22 | 42609415 | C | A | c.1897G>T | p.Ala633Ser | Turkey | M | 15.8 | NA | NA | PM2, PM6, PP3 |
| chr22 | 42609825 | T | C | c.1487A>G | p.Asp496Gly | Iran | M | 24.7 | NA | NA | PM2, PM6, PP3 |
| chr22 | 42609930 | G | A | c.1382C>T | p.Thr461Ile | Portugal | M | 22.1 | NA | NA | PM2, PM6, PP3 |
| chr22 | 42611062 | T | C | c.250A>G | p.Lys84Glu | Kuwait | F | 25.4 | NA | NA | PM2, PM6, PP3 |
| chr22 | 42611091 | G | C | c.221C>G | p.Ser74Cys | Sweden | M | 25.1 | NA | NA | PM2, PM6, PP3 |
| **ZBTB18 NM_205768.2** | | | |  |  |  |  |  |  |  |  |
| chr1 | 244217148 |  | G | c.72C>G | p.Ser24Arg | Lebanon | M | 9.1 | NA | NA | PM2, PM6, PP3 |
| chr1 | 244218178 |  | A | c.1102G>A | p.Val368Ile | Canada | M | 23.5 | NA | 4,07E-06 | PM6, PP3 |
| chr1 | 244218418 |  | T | c.1342A>T | p.Thr448Ser | Kuwait | F | 22.6 | NA | NA | PM2, PM6, PP3 |
| **PDE4A NM_001111307.1** | | | |  |  |  |  |  |  |  |  |
| chr19 | 10557061 |  | A | c.488G>A | p.Arg163Gln | Kuwait | M | 26.3 | yes | 8.39E-06, 1.63E-05 | PS2, PP3 |

**Supplementary Table 3**. Individuals with dual molecular diagnoses

| **Individual** | **Gene** | **Transcript** | **cDNA** | **Protein** | **Zygosity** | **OMIM phenotype** | **OMIM number** | **Mode of inheritance** | **De novo in patient** | **ClinSig** |
| --- | --- | --- | --- | --- | --- | --- | --- | --- | --- | --- |
| A | *FOXG1* | NM_005249.4 | c.256del | p.Gln86Argfs*106 | het | Rett syndrome | 613454 | AD | no | Pathogenic |
|  | *CDK13* | NM_003718.4 | c.1499C>T | p.Thr500Met | het | Congenital Heart Defects, Dysmorphic Facial Features, And Intellectual Developmental Disorder (CHDFIDD) | 603309 | AD | yes | Likely pathogenic |
| B | *TMEM237* | NM_001044385.2 | c.869+1G>A | NA | hom | Joubert syndrome | 614424 | AR | unknown | Pathogenic |
|  | *KMT5B* | NM_017635.4 | c.1180_*1delTAAG | p.Ter394fs | het | Mental Retardation, Autosomal Dominant 51 | 617788 | AD | unknown | Likely pathogenic |

Het: heterozygous, Hom: homozygous, AR: autosomal recessive, AD: autosomal dominant

Additional file 1: **Table S4**. HPO terms listed for all (likely) pathogenic mutation carriers

| cDNA | Protein | HPO terms | HPO terms expanded |
| --- | --- | --- | --- |
| CDK13 NM_003718.4 |  |  |  |
| c.1050delC | p.Tyr351fs | HP:0000708,HP:0001249,HP:0001250,HP:0001263,HP:0002072,HP:0003128 | Behavioral abnormality, Intellectual disability, Seizures, Global developmental delay, Chorea, Lactic acidosis |
| c.1499C>T | p.Thr500Met | HP:0000252,HP:0000486,HP:0000505,HP:0000750,HP:0001249,HP:0001252,HP:0001263,HP:0001270,HP:0001508,HP:0002500,HP:0006808 | Microcephaly, Strabismus, Visual impairment, Delayed speech and language development, Intellectual disability, Muscular hypotonia, Global developmental delay, Motor delay, Failure to thrive, Abnormality of the cerebral white matter, Cerebral hypomyelination |
| c.1630C>T | p.Gln544* | HP:0000717,HP:0000750,HP:0001270,HP:0006989 | Autism, Delayed speech and language development, Motor delay, Dysplastic corpus callosum |
| c.2134G>A | p.Gly712Arg | HP:0001263 | Global developmental delay |
| c.2147A>G | p.Tyr716Cys | HP:0000252,HP:0000750,HP:0001270,HP:0001360,HP:0001363,HP:0001508,HP:0001562,HP:0001999,HP:0003396,HP:0007099 | Microcephaly, Delayed speech and language development, Motor delay, Holoprosencephaly, Craniosynostosis, Failure to thrive, Oligohydramnios,Abnormal facial shape, Syringomyelia,Arnold-Chiari type I malformation |
| c.2525A>G | p.Asn842Ser | HP:0000316,HP:0000369,HP:0000750,HP:0001159,HP:0001249,HP:0001270,HP:0001999 | Hypertelorism, Low-set ears,Delayed speech and language development, Syndactyly, Intellectual disability, Motor delay, Abnormal facial shape |
| c.2525A>T | p.Asn842Ile | HP:0000486,HP:0000750,HP:0001252,HP:0001263,HP:0001270,HP:0001631,HP:0001780,HP:0001999,HP:0002079,HP:0002212,HP:0012825 | Strabismus, Delayed speech and language development, Muscular hypotonia,Global developmental delay, Motor delay, Atrial septal defect, Abnormality of toe, Abnormal facial shape, Hypoplasia of the corpus callosum, Curly hair, Mild |
| CHD4 NM_001273.3 |  |  |  |
| c.1901A>G | p.Lys634Arg | HP:0000750,HP:0000964,HP:0001257,HP:0001263,HP:0001264,HP:0001508,HP:0001762,HP:0002373,HP:0004322 | Delayed speech and language development, Eczema, Spasticity, Global developmental delay, Spastic diplegia, Failure to thrive, Talipes equinovarus, Febrile seizures, Short stature |
| KCNQ3 NM_004519.3 |  |  |  |
| c.688C>T | p.Arg230Cys | HP:0000486,HP:0000750,HP:0000767,HP:0001249,HP:0001252,HP:0001263,HP:0001270,HP:0001763,HP:0001999,HP:0002197,HP:0002376,HP:0002705 | Strabismus, Delayed speech and language development, Pectus excavatum, Intellectual disability, Muscular hypotonia, Global developmental delay, Motor delay, Pes planus,Abnormal facial shape, Generalized seizures, Developmental regression, High, narrow palate |
| c.688C>T | p.Arg230Cys | HP:0000252,HP:0001252,HP:0001263 | Microcephaly, Muscular hypotonia, Global developmental delay |
| KMT5B NM_017635.4 | |  |  |
| c.315_319delTCCTT | p.Pro106fs | HP:0000218,HP:0000319,HP:0000325,HP:0000369,HP:0000378,HP:0000545,HP:0000678,HP:0000750,HP:0001252,HP:0001263,HP:0001270,HP:0001382,HP:0001388,HP:0001508,HP:0001875,HP:0001999,HP:0002019,HP:0002716,HP:0004322,HP:0004325,HP:0100807 | High palate, Smooth philtrum, Triangular face, Low-set ears, Cupped ear, Myopia, Dental crowding, Delayed speech and language development, Muscular hypotonia, Global developmental delay, Motor delay, Joint hypermobility, Joint laxity, Failure to thrive, Neutropenia, Abnormal facial shape, Constipation, Lymphadenopathy, Short stature, Decreased body weight, Long fingers |
| c.347_348delCT | p.Ser116fs | HP:0000194,HP:0000256,HP:0000486,HP:0000750,HP:0001252,HP:0001263,HP:0001270,HP:0001999,HP:0003097,HP:0006119,HP:0006224 | Open mouth, Macrocephaly, Strabismus, Delayed speech and language development, Muscular hypotonia, Global developmental delay, Motor delay, Abnormal facial shape, Short femur, Proximal tapering of metacarpals, Tapering pointed ends of distal finger phalanges |
| c.1180_*1delTAAG (NM_016028.4) | p.Ter394fs | HP:0000505,HP:0000622,HP:0001252,HP:0001263,HP:0006829 | Visual impairment, Blurred vision, Muscular hypotonia, Global developmental delay, Severe muscular hypotonia |
| TCF20 NM_005650.3 |  |  |  |
| c.3440C>T | p.Pro1147Leu | HP:0000147,HP:0001279,HP:0001324,HP:0001634,HP:0002018,HP:0002355,HP:0002592,HP:0004976,HP:0008090,HP:0008441,HP:0010562,HP:0012317,HP:0012531,HP:0012825,HP:0030127,HP:0100512,HP:0100773,HP:0100781 | Polycystic ovaries, Syncope, Muscle weakness, Mitral valve prolapse, Nausea, Difficulty walking, Gastric ulcer, Knee dislocation, Ankylosis of feet small joints, Herniation of intervertebral nuclei, Keloids, Sacroiliac arthritis, Pain, Mild, Endometriosis, Vitamin D deficiency, Cartilage destruction, Abnormality of the sacroiliac joint |
| ZBTB18 NM_205768.2 | |  |  |
| c.1307G>A | p.Arg436His | HP:0000733,HP:0000750,HP:0001249,HP:0001256,HP:0002353,HP:0010850 | Stereotypy, Delayed speech and language development, Intellectual disability, Intellectual disability, mild, EEG abnormality, EEG with spike-wave complexes |
| NFIA NM_001145512.1 | |  |  |
| c.252dupA | p.Tyr85fs | HP:0000750,HP:0001249,HP:0001270,HP:0001363,HP:0010442 | Delayed speech and language development, Intellectual disability, Motor delay, Craniosynostosis, Polydactyly |
| c.1427A>C | p.Asp476Ala | HP:0000316,HP:0000343,HP:0000407,HP:0000457,HP:0000463,HP:0000637,HP:0000750,HP:0001252,HP:0001263,HP:0001270,HP:0001319,HP:0001999,HP:0002705,HP:0002876,HP:0002921,HP:0004952,HP:0005280,HP:0005425,HP:0008070,HP:0008587,HP:0008689,HP:0009909,HP:0011220,HP:0011304,HP:0011359,HP:0012369,HP:0012510,HP:0030875 | Hypertelorism, Long philtrum, Sensorineural hearing impairment, Depressed nasal ridge, Anteverted nares, Long palpebral fissure, Delayed speech and language development, Muscular hypotonia, Global developmental delay, Motor delay,Neonatal hypotonia, Abnormal facial shape, High, narrow palate, Episodic tachypnea, Abnormality of the cerebrospinal fluid, Pulmonary arteriovenous fistulas, Depressed nasal bridge, Recurrent sinopulmonary infections, Sparse hair, Mild neurosensory hearing impairment, Bilateral cryptorchidism, Uplifted earlobe, Prominent forehead, Broad thumb, Dry hair, Abnormality of malar bones, Extra-axial cerebrospinal fluid accumulation, Abnormality of pulmonary circulation |

Additional file 1: **Table S5**. Number of rare, protein-changing variants found in the NDD patients

|  | **All NDD patients (n=4351)** | | | | | |
| --- | --- | --- | --- | --- | --- | --- |
| **Gene** | **Missense** | **Stop gain/lost** | **Splice** | **Frameshift** | **Total** | **Pathogenic/Likely pathogeni** |
| ***CDK13*** | 8 | 1 | 0 | 1 | 10 | 7 |
| ***CHD4*** | 9 | 0 | 0 | 0 | 9 | 1 |
| ***CNOT3*** | 7 | 0 | 0 | 0 | 7 | 0 |
| ***KCNQ3*** | 7 | 0 | 0 | 0 | 7 | 2 |
| ***KMT5B*** | 4 | 0 | 1 | 3 | 8 | 3 |
| ***MSL3*** | 2 | 0 | 0 | 0 | 2 | 0 |
| ***PUF60*** | 2 | 0 | 0 | 0 | 2 | 0 |
| ***QRICH1*** | 5 | 0 | 0 | 0 | 5 | 0 |
| ***SET*** | 1 | 0 | 0 | 0 | 1 | 0 |
| ***TCF20*** | 10 | 0 | 0 | 0 | 10 | 1 |
| ***ZBTB18*** | 4 | 0 | 0 | 0 | 4 | 1 |
| ***CSNK2A1*** | 1* | 0 | 0 | 0 | 1 | 0 |
| ***PPM1D*** | 0 | 0 | 0 | 0 | 0 | 0 |
| ***GNAI1*** | 0 | 0 | 0 | 0 | 0 | 0 |

- Previously published in [[1](#_ENREF_1)]


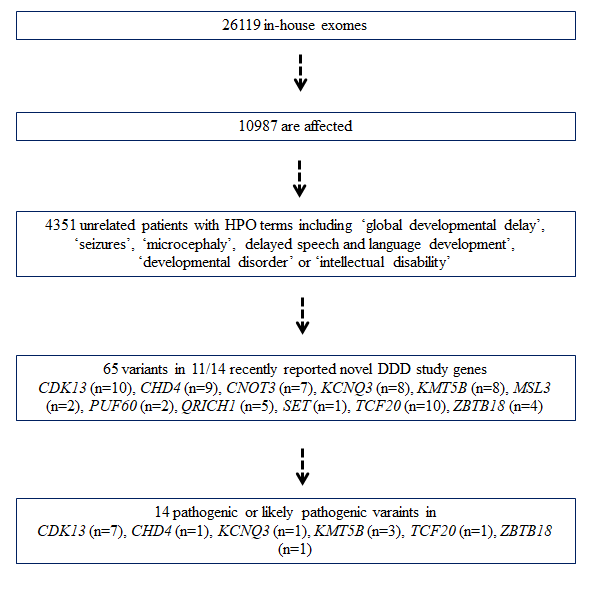


Additional file 1: **Figure S1.** Overview of study: Workflow of identification of 14 (likely) pathogenic variants (6 of 14 candidate genes) in 14 of 4351 patients.

| **Defect in the atrial septum** |  |  |  |  |  |  |  |
| --- | --- | --- | --- | --- | --- | --- | --- |
| **Abnormality of the cerebrum** |  |  |  |  |  |  |  |
| **Cognitive impairment** |  |  |  |  |  |  |  |
| **Abnormal face shape** |  |  |  |  |  |  |  |
| **Holoprosencephaly** |  |  |  |  |  |  |  |
| **Low-set ears** |  |  |  |  |  |  |  |
| **Microcephaly** |  |  |  |  |  |  |  |
| **Feeding difficulties in infancy** |  |  |  |  |  |  |  |
| **Abnormality of the hair** |  |  |  |  |  |  |  |
| **Strabismus** |  |  |  |  |  |  |  |
| **Abnormality of calvarial morphology** |  |  |  |  |  |  |  |
| **Muscular hypotonia** |  |  |  |  |  |  |  |
| **Behavioural abnormalities** |  |  |  |  |  |  |  |
| **Seizures** |  |  |  |  |  |  |  |
| **Delayed speech or language** |  |  |  |  |  |  |  |
| **Global developmental delay** |  |  |  |  |  |  |  |
| **Intellectual disability** |  |  |  |  |  |  |  |
| **Autism** |  |  |  |  |  |  |  |
| **Hypoplasia of the corpus callosum** |  |  |  |  |  |  |  |
|  | **p.Tyr351fs** | **p.Thr500Met** | **p.Gln544*** | **p.Gly712Arg** | **p.Tyr716Cys** | **p.Ash842Ser** | **p.Ash842Ile** |

Additional file 1: **Figure S2**. HPO terms composite for CDK13 pathogenic/likely pathogenic carriers. HPO terms that overlap in different mutation carriers is highlighted in red.

| **Delayed speech and language development** |  |  |  |
| --- | --- | --- | --- |
| **High palate** |  |  |  |
| **Smooth philtrum** |  |  |  |
| **Triangular face** |  |  |  |
| **Low-set ears** |  |  |  |
| **Cupped ear** |  |  |  |
| **Myopia** |  |  |  |
| **Muscular hypotonia** |  |  |  |
| **Global developmental delay** |  |  |  |
| **Motor delay** |  |  |  |
| **Joint hypermobility** |  |  |  |
| **Joint laxity** |  |  |  |
| **Neutropenia** |  |  |  |
| **Abnormal facial shape** |  |  |  |
| **Short stature** |  |  |  |
| **Brain atrophy** |  |  |  |
| **Macrocephaly** |  |  |  |
|  | **p.Pro106fs** | **p.Ser116fs** | **p.Ter394fs** |

Additional file 1: **Figure S3**. HPO terms composite for KMT5B pathogenic/likely pathogenic variant carriers. HPO terms that overlap in different mutation carriers is highlighted in red.
